# Ghrelin for neuroprotection in post-cardiac arrest coma: a 1-year follow-up of cognitive and psychosocial outcomes

**DOI:** 10.1093/ehjacc/zuae119

**Published:** 2024-10-24

**Authors:** Pauline van Gils, Sjoukje Nutma, Karen Meeske, Caroline van Heugten, Walter van den Bergh, Norbert Foudraine, Joost le Feber, Margreet Filius, Michel van Putten, Bert Beishuizen, Jeannette Hofmeijer, for the GRECO investigators, S Nutma, A Beishuizen, W M van den Bergh, N A Foudraine, J le Feber, P M G Filius, A D Cornet, J W Vermeijden, J van der Palen, M J A M van Putten, J Hofmeijer, H B van der Worp, A J C Slooter, M van Smeeden, E Wilms, Martin Rinket, Tim Krol, Rosalie Visser, Esther van Veen, Lucien Gijsbers, Manon Fleuren-Janssen, Michel Kreijtz, Hester Tamminga, Margreet Filius, Martin Rinket, Tim Krol, Wim Addink, Rob Damink, Marlies Snoek-Pecht, Michel Kreijtz, Hester Tamminga, Suzanne Dittrich, Margriet Bosma, Jerôme Appeldoorn, Jolanda Elenbaas, Vera IJmker, Laura de Bever, Ozzy Roesink

**Affiliations:** Department of Clinical Neurophysiology, Technical Medical Center, University of Twente, Drienerlolaan 5, 7522 NB Enschede, The Netherlands; Department of Psychiatry and Neuropsychology, School for Mental Health and Neuroscience, Maastricht University, Universiteitssingel 40, 6229 ER Maastricht, The Netherlands; Limburg Brain Injury Center, Maastricht University, Universiteitssingel 40, 6229 ER Maastricht, The Netherlands; Department of Clinical Neurophysiology, Technical Medical Center, University of Twente, Drienerlolaan 5, 7522 NB Enschede, The Netherlands; Department of Neurology, Medisch Spectrum Twente, Koningstraat 1, 7512 KZ Enschede, The Netherlands; Department of Medical Psychology, Medisch Spectrum Twente, Koningstraat 1, 7512 KZ Enschede, The Netherlands; Limburg Brain Injury Center, Maastricht University, Universiteitssingel 40, 6229 ER Maastricht, The Netherlands; Department of Neuropsychology and Psychopharmacology, Faculty of Psychology and Neuroscience, Maastricht University, Universiteitssingel 40, 6229 ER Maastricht, The Netherlands; Department of Critical Care, University Medical Center Groningen, University of Groningen, Hanzeplein 1, 9713 GZ Groningen, The Netherlands; Department of Critical Care, VieCuri Medical Center, Tegelseweg 210, 5912 BL Venlo, The Netherlands; Department of Clinical Neurophysiology, Technical Medical Center, University of Twente, Drienerlolaan 5, 7522 NB Enschede, The Netherlands; Department of Clinical Pharmacy, Rijnstate Hospital, Wagnerlaan 55, 6815 AD Arnhem, The Netherlands; Department of Clinical Neurophysiology, Technical Medical Center, University of Twente, Drienerlolaan 5, 7522 NB Enschede, The Netherlands; Department of Neurology, Medisch Spectrum Twente, Koningstraat 1, 7512 KZ Enschede, The Netherlands; Department of Critical Care, Medisch Spectrum Twente, Koningstraat 1, 7512 KZ Enschede, The Netherlands; Department of Clinical Neurophysiology, Technical Medical Center, University of Twente, Drienerlolaan 5, 7522 NB Enschede, The Netherlands; Department of Neurology, Rijnstate Hospital, Wagnerlaan 55, 6815 AD Arnhem, The Netherlands

**Keywords:** Cardiac arrest, Acyl-ghrelin, Neuroprotection, Cognitive outcome, Psychosocial outcomes

## Abstract

**Aims:**

Effective treatments to improve brain recovery after cardiac arrest are needed. Ghrelin showed efficacy in experimental models and was associated with lower neuron-specific enolase levels in the clinical Ghrelin in Coma (GRECO) trial. Here, we present cognitive and psychosocial outcomes at 1-year follow-up.

**Methods and results:**

GRECO was a Phase 2 multicentre, double-blind, randomized, placebo-controlled trial in comatose patients after cardiac arrest. The intervention was intravenous acyl-ghrelin 600 μg twice daily or placebo for 1 week, starting within 12 h after the arrest. Patients were assessed after 1 year using cognitive tests and questionnaires measuring participation, health-related quality of life, mood, and caregiver strain. Composite z-scores of the cognitive tests were computed by comparing the scores with those of a norm population and averaging the tests for memory, attention, and executive functioning separately. Groups were compared based on composite z-scores and cut-off scores for psychosocial outcomes. Of the 160 participants originally included, 66 of the 85 participants who survived to 1 year after OHCA completed the psychosocial and cognitive follow-up. The intervention group scored numerically higher across the cognitive domains compared with the control group, but the differences were not statistically significant (memory median = −0.850 vs. −1.385, *U* = 424.5, *P* = 0.587; attention median = −0.733 vs. −0.717, *U* = 420.5, *P* = 0.548; and executive functioning median = −0.311 vs. −0.482, *U* = 408.5*, P = 0.323*). There were significantly fewer signs of depression in the intervention group (*U* = 322.5, *P* = 0.014).

**Conclusion:**

This predefined secondary analysis found that ghrelin treatment was associated with non-significantly but consistently better cognitive outcomes and significantly fewer signs of depression. This is in line with the primary outcomes.

**Clinical trial registration:**

Clinicaltrialsregister.eu: EUCTR2018-000005-23-NL

## Graphical Abstract

**Graphical Abstract:**
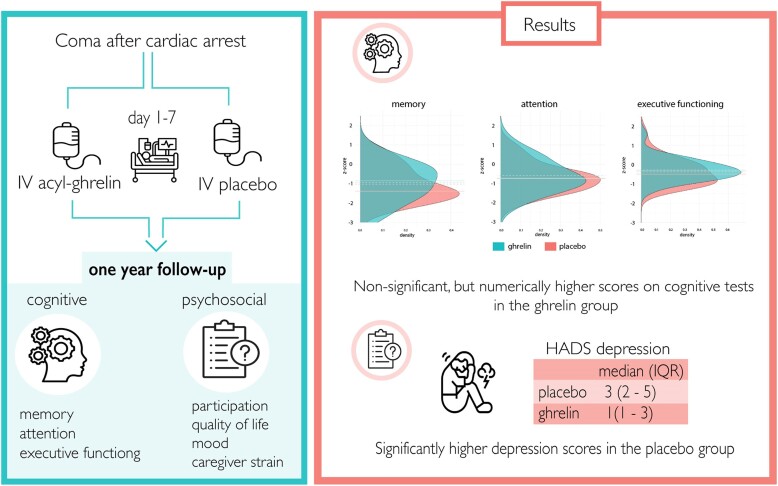

## Introduction

Approximately half of all patients in a comatose state after a cardiac arrest die as a result of severe hypoxic brain injury.^1–3^ The other half, which mostly survives to hospital discharge, is at substantial risk of long-term cognitive impairment.^4^ To improve outcome after cardiac arrest, there is a pressing need for effective treatments to prevent secondary injury and promote brain recovery after a hypoxic insult. More than 20 potential neuroprotective treatments have been tested in clinical trials, but none showed unequivocal evidence of effectiveness.^5^ One of the most promising treatments is mild therapeutic hypothermia, although, despite many randomized trials, there is no indication that TTM provides a clinically meaningful benefit.^6,7^

The recent translational Phase 2 Ghrelin in Coma (GRECO) trial showed safety and potential effectiveness in enhancing neurological outcome of intravenous treatment with acyl-ghrelin during the first 7 days after cardiac arrest.^5^ Results showed a statistically significant reduction of neuron-specific enolase values at Day 1 after cardiac arrest in the intervention group, as well as a non-significant shift towards a better neurological outcome at 6 months that was consistent over all cerebral performance categories (CPCs) and subgroups.

Ghrelin is a naturally occurring hormone that acts as a mildly excitatory neurotransmitter in the brain. Treatment with acylated ghrelin (acyl-ghrelin), the bioactive form, has been associated with better functional recovery of cultured neurons exposed to hypoxia under experimental *in vitro* conditions and improved synapse recovery.^8^ In rat models, ghrelin has shown promising results in preventing neuronal apoptosis after cardiac arrest and improving neurological recovery, especially in the hippocampus.^9,10^ Ghrelin has been administered in healthy participants as well as patients with obesity, diabetes, neurodegenerative diseases, and cardiac failure. In these groups, it has demonstrated an excellent safety profile, with flushing and gastric rumbles as the most common side effects.^11,12^

Mild cognitive impairment is common after OHCA but is not always captured by the CPC score.^13^ Neuropsychological assessment offers more sensitive instruments to identify relatively subtle impairments in cognition, mood, and daily functioning. Identifying those is important, as even mild cognitive impairment after OHCA can lead to reduced daily functioning, societal participation, and quality of life.^14,15^ Still, cognitive and psychosocial outcomes are often neglected as outcomes in trials on treatment of cardiac arrest patients.

The current study is a predefined secondary analysis of the psychosocial and cognitive outcomes of patients included in the GRECO trial at 1-year follow-up. We hypothesized that acyl-ghrelin treatment during post-anoxic coma would be associated with better cognitive functioning at 1-year follow-up, in comparison with placebo treatment. In addition, we hypothesized that acyl-ghrelin treatment would be associated with a more positive outcome on participation, health-related quality of life, mood, and caregiver strain after 1 year compared with placebo. If positive, these results would further support steps towards a Phase 3 trial for the collection of conclusive evidence of effectiveness.

## Methods

### Study design

The GRECO trial was an investigator-initiated Phase 2 multicentre, double-blind, randomized, placebo-controlled trial to assess the effects of acyl-ghrelin treatment on neurological outcome of comatose cardiac arrest patients. Patients were included at three intensive care units in the Netherlands between January 2019 and October 2022, with a follow-up until October 2023. Details on the methodology and the primary outcomes are reported elsewhere.^5^ The current analysis is a predefined secondary analysis of 1-year outcomes of the participants who survived. The trial was approved by the central medical ethics committee (NL64594.044.18) and the research boards of each participating centre. Initial written informed consent was obtained from legal representatives. Additional informed consent of the patients was obtained for the 12-month follow-up assessment.

### Participants

Comatose patients after cardiac arrest and successful resuscitation were eligible for inclusion within 12 h after return of spontaneous circulation. The inclusion criteria were an age of 18 years or older and haemodynamic and respiratory stability, as determined by the treating physician. Exclusion criteria included a known progressive neurological disease and expected death within 48 h.

### Treatment

Patients were randomly assigned in a 1:1 ratio to receive acyl-ghrelin (intervention group) or placebo (control group). Treating and assessing clinicians, and patients, were blinded for group assignment in this placebo-controlled clinical trial. The investigational treatment was intravenous acyl-ghrelin, 600 μg dissolved in 50 cc normal saline, infused in 30 min, twice daily for 1 week, starting within 12 h after cardiac arrest. Patients in the control group received a placebo (50 cc normal saline without acyl-ghrelin). Study medication was stopped only in case of pending death or discharge to home and was additional to standard care by the discretion of the treating team, generally following the European guidelines for comatose patients after cardiac arrest, including targeted temperature treatment at 36°C.^16^ Decisions regarding withdrawal of treatment were based on the Dutch guideline, which is largely in line with the European guideline.^16^ Withdrawal of treatment could be considered during normothermia and while the patient was not receiving sedation, if there was an incomplete return of brain stem reflexes, a malignant EEG pattern at ≥24 h after cardiac arrest, or bilateral absence of N20 somatosensory evoked potentials.

### Outcome measures

The primary outcome of this paper was the performance on the domains of memory, attention, and executive functioning on the cognitive assessment at 1-year follow-up. The primary outcome of the trial (CPC at 6 months) and safety outcomes have been reported elsewhere.^5^ Secondary outcome measures included questionnaires on societal participation, health-related quality of life, mood, and caregiver strain 12 months after cardiac arrest. For collection of these outcomes, patients were invited to the hospital or visited at their place of residence.

### Cognitive assessment

Cognitive assessment at 1-year follow-up consisted of the Montreal cognitive assessment (MoCA), the Stroop colour and word test (Stroop), the Rey Auditory Verbal Learning Test (RAVLT), the trail making test (TMT), the Raven, and the letter fluency. The cognitive assessment took approximately 1.5 h. More detailed descriptions of the neuropsychological tests can, among other sources, be found in the handbook of neuropsychological assessment.^17^

The Stroop is a measure of processing speed and executive functioning.^18^ It consists of three parts. The first two parts assess the speed of information processing, and the third part measures response inhibition. Longer completion time of the Stroop indicates poorer performance. The interference score is obtained by subtracting the mean of Stroops 1 and 2 from Stroop 3.

The RAVLT is a memory test. It measures immediate recall, delayed recall, and recognition.^19^ The score on immediate recall ranges from 0 to 75, the delayed recall ranges from 0 to 15, and the recognition score ranges from 0 to 30. Higher scores indicate better performance.

*Trail making test AB* is a measure of psychomotor and processing speed (Part A) and executive functioning (Part B).^20^ The interference score, assessing executive functioning, is the completion time of Part B, corrected for the psychomotor speed obtained in Part A. Longer completion time indicates poorer performance.

The *letter fluency* is used to measure executive functioning. Participants have to produce words beginning with a specific letter within 1 min.^21^ Higher scores indicate better performance.

*Nine-item form of the Raven’s Advanced Progressive Matrices* (RAVEN) is used to measure the level of abstract reasoning.^22^ Scores range from 0 to 9; higher scores indicate better performance.

The MoCA is a screening instrument to identify overall cognitive impairment.^23^ Higher scores indicate better performance, with scores ranging from 0 to 30. A correction for educational level was applied (one extra point to those with 12 or fewer years of formal education). Scores < 26 are considered cognitively impaired.^23,24^

The following questionnaires were administered, taking approximately 30 min:

The *Hospital Anxiety and Depression Scale* (HADS) was used to assess depression and anxiety.^25^ A higher score represents more complaints: 0–7 indicates no anxiety or depression, 8–10 indicates a possible anxiety disorder or depression, and a score of 11–21 indicates a probable anxiety disorder or depression.^26^

The *Utrecht Scale for Evaluation of Rehabilitation-Participation* (USER-P) was used as a measure of participation.^27^ It consists of 31 items, and it assesses three aspects of participation: frequency, experienced restrictions, and satisfaction. A higher score indicates a more favourable outcome.

The *caregiver strain index* (CSI) was used to estimate strain among family caregivers.^28^ It consists of 13 yes (1) or no (0) questions, with higher total score indicating more strain. A score of 0–6 indicates ‘no strain’, 7–10 ‘strain’, and 11–13 ‘heavy strain’.^29^

The *EuroQol 5 Dimensions 5 levels* (EQ-5D-5L) was used to measure health-related quality of life.^30^ The first part consists of five questions assessing mobility, self-care, usual activities, pain/discomfort, and anxiety/depression with scores each ranging from 1 (not a problem) to 5 (an extreme problem). The scores were converted into index values that can range between 0 and 1, with higher scores indicating a better health status.^31^ The second part is a visual analogue scale of 0 (worst imaginable health) to 100 (best imaginable health).

### Statistical analysis

The statistical analysis plan was finalized before the database was locked, and data were analysed. Data analyses were performed using SPSS Statistics (version 27). Differences in demographic characteristics were tested with independent *t*-tests. Normality for all dependent variables was checked with the Shapiro–Wilk test. Z-scores were calculated for the cognitive tests to control for age, education level, and sex using the norms of the Dutch Association of Psychologists 2012 and Maasnorms.^32–36^ Higher z-scores indicate better performance for all tests. To prevent outliers of having an unreasonable effect on the composite z-scores, a maximum of 3 standard deviations and a minimum of −3 standard deviations were implemented for the z-scores on individual tests, including 99.7% of a normally distributed population. We calculated a composite score for each cognitive domain by averaging the z-scores of the tests that assessed that same domain:

- Executive functioning: Stroop interference score, TMT interference score, fluency, Raven, Stroop 3, and TMT B
- Memory: RAVLT encoding, RAVLT delayed recall, and RAVLT recognition
- Attention: Stroop 1, Stroop 2, and TMT A

Median differences in composite scores in the three domains and the questionnaires between the intervention and control groups were analysed using Mann–Whitney *U* tests. No correction for multiple testing was applied. For questionnaires with a cut-off score, the percentage of participants exceeding that threshold was calculated.

## Results

### Demographics

**Figure 1:**
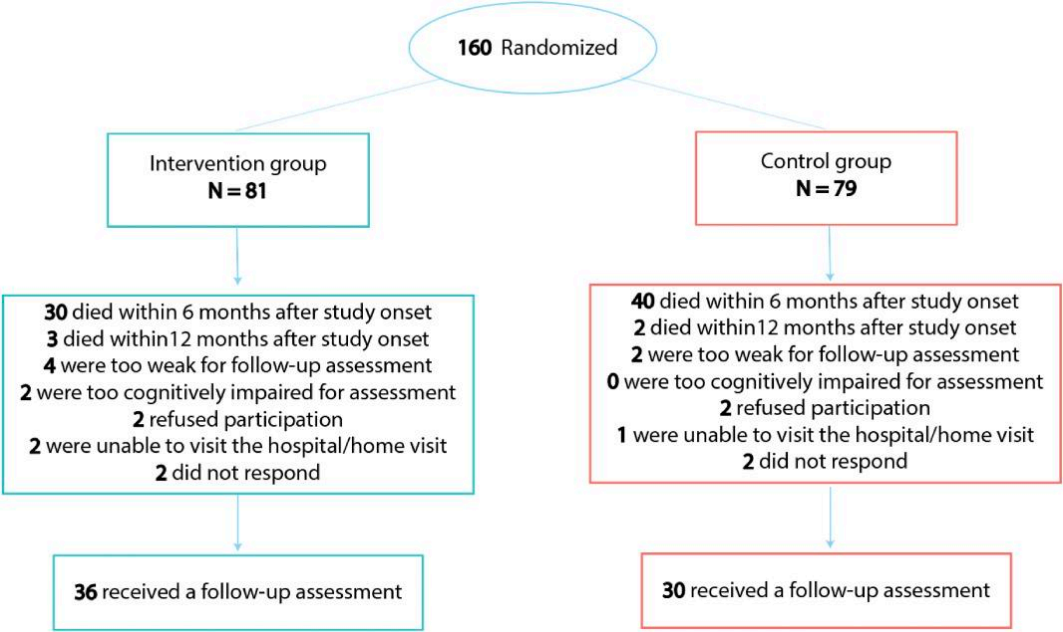
Flow diagram of patients from inclusion to follow-up assessment.

Of the 160 participants originally included in the GRECO trial, 66 of the 85 participants who survived to 1 year after OHCA participated in the 12-month follow-up (*Figure 1*). *Table 1* shows the demographic and cardiac arrest characteristics of the participants. None of these characteristics significantly differed between the intervention and control groups.

**Table 1:** Demographic and cardiac arrest characteristics of the intervention and control group in *N* (%) or mean (SD)

Characteristic	Ghrelin (*n* = 36)	Placebo (*n* = 30)
Age in years, mean (SD)	63 (14)	65 (12)
Range	27–89	39–84
Sex (% male)	26 (72)	24 (80)
Level of education^a^, *n* (%)		
Low	5 (14)	3 (10)
Middle	21 (58)	18 (60)
High	7 (19)	7 (23)
Missing	3 (8)	2 (7)
ROSC in minutes, mean (SD)	17 (15)	15 (9)
Range	5–90	2–50
Initial rhythm, *n* (%)		
VF	29 (81)	23 (77)
VT	3 (8)	1 (3)
PEA	0 (0)	3 (10)
Unknown	4 (11)	3 (10)
CPC score 12 months, *n* (%)		
1	18 (67)	16 (70)
2	8 (30)	6 (26)
3	1 (3)	1 (4)
CPC score 6 months, *n* (%)		
1	21 (52)	17 (57)
2	10 (29)	9 (30)
3	3 (9)	4 (13)
NSE levels^b^, mean (SD)	21 (9.5)	29 (20.1)

ROSC, return to spontaneous circulation; VF, ventricular fibrillation; VT, ventricular tachycardia; PEA, pulseless electrical activity; SD, standard deviation.

^a^Level of education was classified with the Verhage levels of education (Verhage, 1983).

^b^NSE levels were measured at a median time of 21 h after cardiac arrest.

### General cognitive functioning

There was no difference in MoCA scores between the intervention and control group (median = 26.0 vs. 26.0, *U* = 501, *P* = 0.721). In the intervention group, 14 (41.2%) participants scored below the cut-off score of 26 compared with 13 (46.4%) participants in the control group.

### Cognitive domain assessment

**Figure 2:**
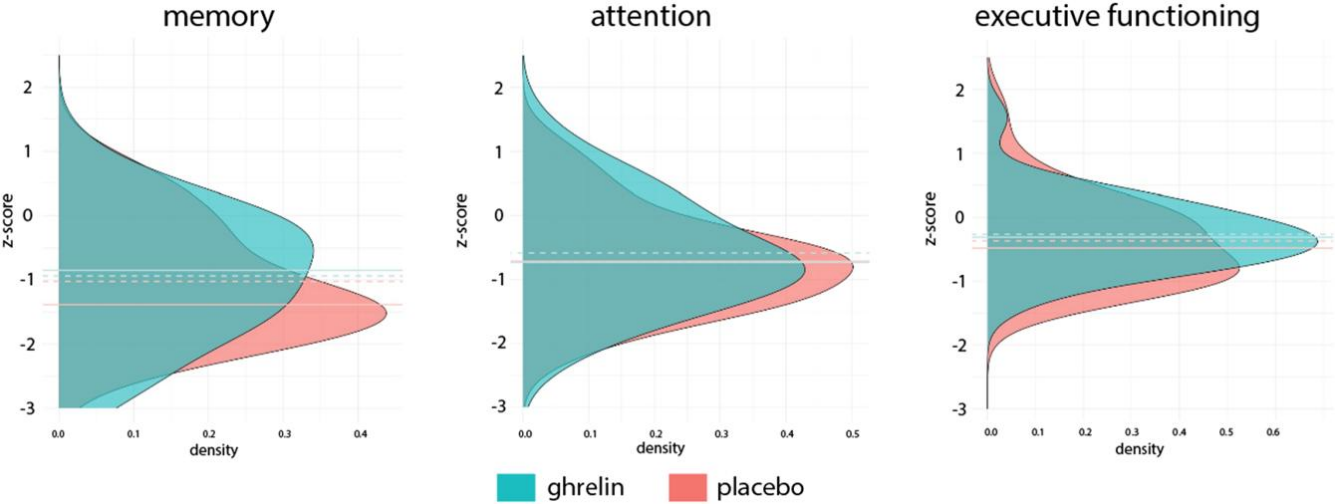
Density plots of the composite z-scores of the cognitive domains for the intervention and control group; the dotted lines indicate means and the solid lines indicate medians.

The medians and z-scores of the cognitive tests and cognitive domains can be found in *Table 2* [for means (SD), mean difference scores (95% CIs), and composite scores stratified by CPC score, see Supplementary material online, *Appendix S2*)]. All median composite z-scores were below zero, suggesting worse performance than the average of the general norm population. The composite z-scores of the cognitive domains were generally higher for the intervention group than for the control group, but the differences were not statistically significant (memory median = −0.850 vs. −1.385, *U* = 424.5, *P* = 0.587; attention median = −0.733 vs. −0.717, *U* = 420.5, *P* = 0.548; and executive functioning median = −0.311 vs. −0.482, *U* = 408.5, *P =* 0.323). The distribution of the scores is visualized in *Figure 2*.

**Table 2:** Median raw scores and z-scores of the cognitive tests for the intervention and control group

			Ghrelin (*n* = 36)			Placebo (*n* = 30)	
Domain	Test	*n*	Median (IQR)	z-score	*n*	Median (IQR)	z-score
—	MoCA	34	26.0 (23.8–27.0)	—	28	26.0 (23.0–27.0)	—
	RAVLT						
M	Encoding	33	32.0 (24.5–40.0)^a^	−1.38	29	27.0 (21.5–35.5)^a^	−1.48
M	Recall	33	6.0 (4.0–9.0)^a^	−0.94	29	5.0 (3.0–8.5)	−1.17
M	Recognition	29	28.0 (26.0–30.0)^a^	−0.25	27	28.0 (25.0–30.0)	−0.27
	Stroop (in sec)						
A	Card 1	33	51.0 (46.0–59.5)^a^	−1.10	29	56.0 (49.5–64.0)^a^	−1.39
A	Card 2	33	64.0 (57.0–73.0)^a^	−0.72	29	67.0 (62.5–80.5)^a^	−0.97
EF	Card 3	33	116.0 (92.0–133.5)^a^	−0.42	29	115.0 (89.5–139.5)^a^	−0.38
EF	Interference	33	—	0.18	28	—	0.43
	TMT (in sec)						
A	TMT-A	34	34.5 (29.0–50.0)^a^	0.07	29	34.0 (30.0–43.0)	0.16
EF	TMT-B	33	90.0 (75.5–109.0)	−0.20	29	101.0 (78.5–135.0)	−0.54
EF	Interference	33	—	−0.23	28	—	−0.69
EF	Raven	33	5.0 (3.0–5.5)^a^	−0.59	29	4.0 (3.0–5.5)^a^	−0.62
EF	Fluency	24	34.0 (23.3–42.0)^a^	−0.30	21	34.0 (24.5–37.5)	−0.44
Composite z-scores							*P*-value
Memory		33	−0.85 (−1.67–−0.15)		28	−1.39 (−1.65–−0.33)	0.587
Attention		33	−0.73 (−1.2–−0.08)^a^		28	−0.72 (−1.3–−0.41)^a^	0.548
Executive functioning		33	−0.31 (−0.71–0.09)		29	−0.48 (−0.94–0.08)	0.323

M, memory; A, attention; EF, executive functioning; RAVLT, Rey Auditory Verbal Learning Test; TMT, trail making test; Raven, the Rey Auditory Verbal Learning Test; BNT, the short form of the Boston Naming Test; IQR, interquartile range.

^a^This variable was normally distributed.

### Questionnaires

*Table 3* presents the results of the questionnaires on mood, health-related quality of life, participation, and caregiver strain in the intervention and control group. There were significantly fewer signs of depression in the intervention group than in the control group (*U* = 311.5, *P* = 0.014). The number or patients exceeding the cut-off score for depression was the same in both groups. Other between-group differences were not statistically significant. The intervention group scored more favourably on most (four to eight out of nine) measures.

**Table 3:** Median (IQR) scores on the questionnaires administered at 1 year after cardiac arrest for the intervention and control group

		Ghrelin (*n* = 36)			Placebo (*n* = 30)		
Questionnaire	*n*	Median (IQR)	Exceeds cut-off	*n*	Median (IQR)	Exceeds cut-off	*P-*value^a^
HADS	36			27			
Anxiety		3.0 (0.0–4.7)	3 (8.3%)^d^		3.0 (1.0–6.0)	3 (11.1%)^d^	0.400
Depression		1.0 (1.0–3.0)	2 (5.6%)^d^		3.0 (2.0–5.0)	2 (7.4%)^d^	0.014
Total		4.0 (2.0–8.0)			6.0 (4.0–12.0)		0.078
EQ-5D-5L	35			26			
Index value		0.89 (0.8–1.0)			0.87 (0.7–1.0)		0.530
VAS score		80.0 (75.0–90.0)			80.0 (68.8–90.0)		0.446
USER-P							
Frequency	36	35.2 (27.6–40.6)^b^		26	31.1 (23.3–38.9)^b^		0.134
Limitations	35	96.3 (76.7–100.0)		25	96.7 (90.0–100.0)		0.725
Satisfaction	35	77.8 (72.5–90.6)		25	77.8 (67.8–90.8)^b^		0.752
CSI	18	2.0 (0.0–3.5)	1 (5.6%)^c^	12	2.0 (0.0–3.0)	2 (16.7%)^c^	0.983

HADS, Hospital Anxiety and Depression Scale; USER-P, Utrecht Scale for Evaluation of Rehabilitation-Participation; CSI, caregiver strain index; EQ-5D-5L, The EuroQol 5 Dimensions 5 levels; IQR, interquartile range.

^a^ *P*-values are uncorrected and refer to group score differences assessed with Mann–Whitney *U* tests.

^b^This variable was normally distributed.

^c^Scores > 6 were indicative of caregiver strain.

^d^Scores > 7 were indicative of possible anxiety/depression.

## Discussion

In this predefined secondary analysis of outcomes at 1 year after cardiac arrest of the multicentre randomized GRECO trial, treatment with acyl-ghrelin was associated with fewer signs of depression. There were no significant differences in cognition, societal participation, health-related quality of life, and caregiver strain, but most scores were numerically more favourable in the intervention group.

This was the first Phase 2 trial on the cognitive and psychosocial outcomes on acyl-ghrelin treatment after cardiac arrest. Most of the differences between the intervention and control group were not statistically significant. However, point estimates in the primary analysis and the current predefined secondary analyses are in the same direction and favour the intervention.^5^ Although we did no formal power calculation for the secondary (psychosocial) outcomes, we assume that the lack of statistical difference could be a false negative resulting from insufficient statistical power.

The difference in depression score on the HADS between the groups exceeds the minimum clinically important difference of 1.7 established in patients with cardiovascular disease.^37^ However, given the potential influence of many other psychosocial factors on depression over a year, it is uncertain how much of the difference can be attributed to the ghrelin intervention. While there is no consensus on determining the minimum clinically important differences for combined measures, the point estimates favour the intervention group, suggesting that the combined effect could be clinically relevant. Despite the small differences in test scores, their cumulative impact may hold substantial clinical relevance for cognitive and psychosocial functioning. This is particularly important since our safety analysis showed limited evidence for harmful effects.^5^

Ghrelin treatment shows beneficial effects on memory in rodent studies.^38^ Also, after treatment with ghrelin in cultured neuronal networks under experimental *in vitro* conditions, there were increases in electrophysiological activity and decreases in microscopic measures of apoptosis and synaptic loss after a hypoxic insult compared with untreated networks.^39,40^ Studies in *in vivo* cardiac arrest animal models showed similar results, as well as improved functional recovery.^9^

Strengths of this study include the prospective, double-blind, placebo-controlled, randomized trial design with predefined outcome measures. The major limitation is the relatively small sample size, which is inherent to its Phase 2 trial nature and further impacted by the high mortality rate before follow-up. Second, we cannot fully exclude baseline differences, although there were no statistically significant differences in patient characteristics.^5^ Notably, we lack pre-cardiac arrest measurements of cognition, so it is unknown if functioning before cardiac arrest was comparable between the groups. However, the level of education did not differ.

## Conclusions

In this predefined secondary analysis of the randomized GRECO trial, treatment with acyl-ghrelin was not associated with better cognitive performance as compared with placebo. The treatment was associated with fewer signs of depression. No significant differences were found in participation, caregiver strain, anxiety, and health-related quality of life 1 year after cardiac arrest. However, numeric differences favoured the intervention group and were in line with the primary analysis of neurological outcome at 6 months, as well as with blood biomarker analyses.

## Supplementary Material

zuae119_Supplementary_Data

## Data Availability

The data underlying this article will be shared on reasonable request by the primary investigator of the GRECO trial.
