# Supplementary material for: Ghrelin for neuroprotection in post-cardiac arrest coma: a 1-year follow-up of cognitive and psychosocial outcomes
Source: Eur Heart J Acute Cardiovasc Care. 2024 Oct 24;14(1):5–11. doi: 10.1093/ehjacc/zuae119 (PMC11783279; doi:10.1093/ehjacc/zuae119)
Supplement: zuae119_Supplementary_Data [file zuae119_supplementary_data.zip › van Gils_appendix B_102024.docx]

APPENDIX B

**Table 1**

Mean raw scores (SD) of the cognitive tests for the total, ghrelin intervention, and placebo group.

|  | Ghrelin (n = 36) | |  | Placebo (n = 30) | |  | Total (n = 66) | |
| --- | --- | --- | --- | --- | --- | --- | --- | --- |
|  |  |  |  |  |  |  |  |  |
| Test | n | mean (SD) |  | n | mean (SD) |  | n | Mean (SD) |
| **MoCA** | 34 | 24.68 (4.69) |  | 28 | 24.50 (4.49) |  | 62 | 24.60 (4.56) |
| **RAVLT** |  |  |  |  |  |  |  |  |
| encoding | 33 | 32.09 (9.37) |  | 29 | 30.00 (9.97) |  | 62 | 31.11 (9.63) |
| recall | 33 | 6.39 (3.21) |  | 29 | 5.66 (3.47) |  | 62 | 6.05 (3.33) |
| recognition | 29 | 27.66 (2.47) |  | 27 | 27.26 (2.88) |  | 56 | 27.46 (2.66) |
| **Stroop (in sec)** |  |  |  |  |  |  |  |  |
| card 1 | 33 | 52.61 (10.25) |  | 29 | 56.83 (11.14) |  | 62 | 54.58 (10.80) |
| card 2 | 33 | 66.06 (12.20) |  | 29 | 71.34 (13.43) |  | 62 | 68.53 (12.96) |
| card 3 | 33 | 112.64 (27.89) |  | 29 | 119.86 (39.14) |  | 62 | 116.02 (33.54) |
| **TMT (in sec)** |  |  |  |  |  |  |  |  |
| TMT-A | 34 | 47.50 (53.00) |  | 29 | 39.48 (19.26) |  | 63 | 43.81 (41.00) |
| TMT-B | 33 | 101.73 (56.47) |  | 29 | 117.69 (62.71) |  | 62 | 109.19 (59.52) |
| **Raven** | 33 | 4.33 (1.59) |  | 29 | 4.28 (1.56) |  | 62 | 4.31 (1.56) |
| **Fluency** | 24 | 32.92 (11.55) |  | 21 | 31.76 (15.01) |  | 45 | 32.38 (13.13) |

RAVLT = Rey Auditory Verbal Learning Test; TMT = Trail Making Test; Raven = The Rey Auditory Verbal Learning Test; BNT = The short form of the Boston Naming Test

**Table 2**

*Mean (SD) composite z-scores for the total, ghrelin intervention, and placebo group, including difference scores and 95% confidence intervals.*

|  |  | Ghrelin |  |  | Placebo |  |  | Total |  |
| --- | --- | --- | --- | --- | --- | --- | --- | --- | --- |
|  | N | Mean (SD) |  | N | Mean (SD) |  | N | Mean (SD) | Mean difference ^a^ (95% CI) |
| memory | 33 | -.94 (.96) |  | 28 | -1.02 (.87) |  | 61 | -.98 (.91) | -.09 (-.56, .39) |
| attention | 33 | -.58 (.85) |  | 28 | -.73 (.76) |  | 61 | -.65 (.81) | -.15 (-.56, .27) |
| executive functioning | 33 | -.27 (.56) |  | 29 | -.37 (.70) |  | 62 | -.31 (.62) | -.10 (-.42, .22) |

^a^ Difference scores were obtained by subtracting the mean ghrelin composite z-score from the mean placebo composite z-score.

**Table 3**

*Mean (SD) scores on the questionnaires administered at one year after cardiac arrest for the total, intervention, and control group.*

|  | Ghrelin (n = 36) | |  | Placebo (n = 30) | |  | Total (n = 66) | |
| --- | --- | --- | --- | --- | --- | --- | --- | --- |
|  |  |  |  |  |  |  |  |  |
| Questionnaire | n | mean (SD) |  | n | mean (SD) |  | n | Mean (SD) |
| **HADS** | 36 |  |  | 27 |  |  | 63 |  |
| anxiety |  | 3.17 (3.07) |  |  | 3.93 (3.63) |  |  | 3.49 (3.31) |
| depression |  | 2.44 (3.25) |  |  | 3.74 (3.22) |  |  | 3.00 (3.27) |
| total |  | 5.61 (5.76) |  |  | 7.81 (6.44) |  |  | 6.56 (6.11) |
| **5Q-5D-5L** | 35 |  |  | 26 |  |  | 61 |  |
| index value |  | .88 (.13) |  |  | .83(.21) |  |  | .86 (.17) |
| VAS-score |  | 80.40 (15.13) |  |  | 77.00 (16.02) |  |  | 78.95 (15.48) |
| **USER-P** |  |  |  |  |  |  |  |  |
| Frequency | 36 | 33.54 (9.94) |  | 26 | 29.88 (9.61) |  | 62 | 32.01 (9.89) |
| limitations | 35 | 86.91 (19.17) |  | 25 | 92.30 (10.74) |  | 60 | 89.16 (16.30) |
| Satisfaction | 35 | 78.23 (18.07) |  | 25 | 78.98 (14.24) |  | 60 | 78.54 (16.46) |
| **CSI** | 18 | 2.39 (2.28) |  | 12 | 2.92 (3.75) |  | 30 | 2.60 (2.91) |

HADS: Hospital anxiety and depression scale; USER-P: Utrecht Scale for Evaluation of Rehabilitation-Participation; CSI: caregiver strain index; EQ-5D-5L: The EuroQol 5 Dimensions 5 levels; IQR: interquartile range

**Table 4**

*Medians (IQR) of the composite z-scores stratified by CPC score at 12 months.*

|  | CPC 1 | |  | CPC 2 | |  | CPC 3 | |
| --- | --- | --- | --- | --- | --- | --- | --- | --- |
| Composite  z-scores | Ghrelin  (n = 18) | Placebo  (n = 16) |  | Ghrelin  (n = 7) | Placebo  (n = 6) |  | Ghrelin (n = 0) | Placebo (n = 1) |
| Memory | -.94 (-1.84 - .05) | -.1.43 (-1.19 - -.62) |  | -1.35 (-1.83 - -.17) | -.65 (-.53 - .56) |  | - | -2.27^a^ |
| Attention | -.58 (-.91 - .13) | -.68 (-1.30 - -0.41) |  | -1.00 (-1.53 - .07) | -.68 (-1.11 – 0.00) |  | - | -1.83^a^ |
| Executive functioning | -.32 (-.68 - .02) | -.22 (-1.00 - .21) |  | -.40 (-.76 - .15) | -.44 (-.91 - .27) |  | - | -.27^a^ |

^a^Sample size was too small to compute interquartile ranges

**Table 5***Medians (IQR) of the composite z-scores stratified by CPC score at 6 months.*

|  | CPC 1 | |  | CPC 2 | |  | CPC 3 | |
| --- | --- | --- | --- | --- | --- | --- | --- | --- |
| Composite z-scores | Ghrelin  (n = 19) | Placebo  (n = 17) |  | Ghrelin  (n = 10) | Placebo  (n = 9) |  | Ghrelin  (n = 2) | Placebo (n = 3) |
| Memory | -.73 (-1.25 - .05) | -1.12 (-1.50 - .02) |  | -1.48 (-2.07 - -.45) | -1.39 (-1.64 - -.47) |  | -.54^a^ | -1.92^a^ |
| Attention | -.67 (-.87 - .33) | -.77 (-1.30 - -.21) |  | -1.23 (-1.68 - -.13) | -.73 (-1.27 - -.12) |  | -.27^a^ | -.43^a^ |
| Executive functioning | -.25 (-.78 - .24) | -.64 (-1.03 - .14) |  | -.35 (-.60 - -.11) | -.24 (-.89 - -.04) |  | -.49^a^ | -.27^a^ |

^a^Sample size was too small to compute interquartile ranges
