# Supplementary material for: Ghrelin for neuroprotection in post-cardiac arrest coma: a 1-year follow-up of cognitive and psychosocial outcomes
Source: Eur Heart J Acute Cardiovasc Care. 2024 Oct 24;14(1):5–11. doi: 10.1093/ehjacc/zuae119 (PMC11783279; doi:10.1093/ehjacc/zuae119)
Supplement: zuae119_Supplementary_Data [file zuae119_supplementary_data.zip › vanGils_supplementary A_05072024.docx]

# Supplementary A

# List of GRECO investigators and affiliations

S. Nutma^1,2^, A. Beishuizen^3^, W.M. van den Bergh^4^, , N.A. Foudraine^5^, J. le Feber^1^, P.M.G. Filius^6^, A.D. Cornet^3^, J.W. Vermeijden^3^, J. van der Palen^7,8^, M.J.A.M. van Putten^1,2^, J. Hofmeijer^1,9^

^1^ Department of Clinical Neurophysiology, Technical Medical Center, University of Twente, Enschede, the Netherlands

^2^ Department of Neurology, Medisch Spectrum Twente, Enschede, the Netherlands

^3^ Department of Critical Care, Medisch Spectrum Twente, Enschede, the Netherlands

^4^ Department of Critical Care, University Medical Center Groningen, University of Groningen, Groningen, the Netherlands

^5^ Department of Critical Care, VieCuri Medical Center, Venlo, the Netherlands

^6^ Department of Clinical Pharmacology, Rijnstate Hospital, Arnhem, the Netherlands

^7^ Department of Epidemiology, Medisch Spectrum Twente, Enschede, the Netherlands

^8^ Section Cognition, Data and Education, Faculty of Behavioral, Management and Social Sciences, University of Twente, Enschede, Netherlands

^9^ Department of Neurology, Rijnstate Hospital, Arnhem, the Netherlands

# Acknowledgements

The GRECO trial was funded by a grant from ZonMw and the Hersenstichting. We thank the following for their help and advice.

*Data Safety and Monitoring Board*

Prof. dr. H.B, van der Worp, department of Neurology, University Medical Centre Utrecht, the Netherlands (chair)

Prof. dr. A.J.C. Slooter, department of Intensive Care, University Medical Centre Utrecht, the Netherlands (member)

Dr. M. van Smeeden, department of Epidemiology, University Medical Centre Utrecht, the Netherlands (member)

Dr. E. Wilms, department of Clinical Pharmacology, Pharmacy Hague Hospitals and Haga Hospital, the Netherlands (member)

*Research nurses*

Martin Rinket, Tim Krol (Department of Intensive Care, Medisch Spectrum Twente, Enschede, the Netherlands), Rosalie Visser, Esther van Veen (Department of Neurology, Medisch Spectrum Twente, Enschede, the Netherlands)

Lucien Gijsbers, Manon Fleuren-Janssen (Department of Intensive Care, VieCuri Medical Centre, Venlo, the Netherlands)

Michel Kreijtz, Hester Tamminga (Department of Intensive Care, University Medical Centre Groningen, Groningen, the Netherlands)

*Study medication central storage and distribution*

Margreet Filius (Department of Clinical Pharmacology, Rijnstate Hospital, Arnhem, the Netherlands)

*Study medication local storage and preparation for administration*

Martin Rinket, Tim Krol, Wim Addink, Rob Damink, Marlies Snoek-Pecht (Department of Intensive Care, Medisch Spectrum Twente, Enschede, the Netherlands)

Michel Kreijtz, Hester Tamminga (Department of Intensive Care, University Medical Centre Groningen, Groningen, the Netherlands)

Suzanne Dittrich, Margriet Bosma (Department of Clinical Pharmacology, VieCuri Medical Centre, Venlo, the Netherlands)

Jerôme Appeldoorn (Department of Clinical Pharmacology, University Medical Centre Groningen, Groningen, the Netherlands)

*Monitoring*

Jolanda Elenbaas, Vera IJmker, Department of Epidemiology, Rijnstate Hospital, Arnhem, the Netherlands

*Data Entry and follow-up*

Laura de Bever (Department of Neurology, Medisch Spectrum Twente, Enschede, the Netherlands

Ozzy Roesink (Public Health studies, University of Twente, Enschede, the Netherlands)
